# *Brucella* Shunt Infection as a Rare Presentation of Neurobrucellosis

**DOI:** 10.1155/2019/7291482

**Published:** 2019-01-21

**Authors:** Fatemeh Mehrabian, Zahra Abdi Layaee, Zahra Ahmadinejad

**Affiliations:** ^1^Internal Medicine Resident, Imam Khomeini Hospital Complex, Tehran University of Medical Sciences, Tehran, Iran; ^2^Infectious Diseases Specialist, Department of Infectious Diseases, Imam Khomeini Hospital Complex, Tehran University of Medical Sciences, Tehran, Iran; ^3^Infectious Diseases Specialist, Full Professor, Liver Transplantation Research Center, Imam Khomeini Hospital Complex, Tehran University of Medical Sciences, Tehran, Iran

## Abstract

Brucellosis, as a systemic infection with potential to involving virtually all organ systems, is an endemic zoonosis in Iran. This is the history of a 17-year-old boy with a ventriculoperitoneal (VP) shunt who presented with prolonged fever, constipation, and abdominal pain. Laboratory studies, including cerebrospinal fluid (CSF) and blood tests, revealed a VP shunt infection with *Brucella* spp. We treated the patient with rifampicin, trimethoprim-sulfamethoxazole (cotrimoxazole), and ceftriaxone. Also, the shunt was temporarily replaced with an extraventricular drain until the CSF culture was sterile and a new permanent VP shunt could be inserted. We report this case to underline the diagnostic possibility of brucellosis in every case of protracted fever of unknown origin (FUO), irrespective of accompanying signs and symptoms.

## 1. Introduction

Brucellosis is an endemic zoonosis in the countries of Persian Gulf including Iran [1, 2]. It is a systemic infection with multiorgan involvement [2]. Among many variable presentations are the neurologic ones which are well gathered under the umbrella of “neurobrucellosis.” It occurs in 2–7 percent of affected individuals, and nervous system manifestations include encephalitis, meningitis, myelitis, radiculitis, and/or neuritis (with involvement of cranial or peripheral nerves) [3]. However, a much less described subtype of neurobrucellosis is ventriculoperitoneal (VP) shunt infection [2] which is the subject of this case report.

## 2. Case Report

A 17-year-old boy living in a care home due to his cerebral palsy was referred to “Imam Khomeini Hospital Complex”—a tertiary care educational hospital in Tehran, Iran—with a two-month history of fever, abdominal pain, and constipation. A VP shunt had been inserted for him at age four for treating hydrocephalus and subsequent refractory seizures. His recent symptoms had an intermittent pattern subsiding transiently with symptomatic therapies such as antipyretics and laxatives. Empiric antibiotics too, including parenteral ceftriaxone, were prescribed for the patient several times, but no response was achieved.

At presentation, the physical exam revealed a blood pressure of 90/60 mmHg, a pulse rate of 100/min, a temperature of 38.5°C, and generalized tenderness in the abdominal palpation. The initial laboratory findings revealed increased erythrocyte sedimentation rate (ESR) and C-reactive protein (CRP) and a mild leukocytosis (Table 1).

Although physical examination was not compatible with peritonitis, we asked for a confirmatory abdominal X-ray which was unrevealing. The abdominal ultrasound done for more evaluation of pain showed a pseudocyst exactly at the distal end of the VP shunt. To evaluate the shunt infection, cerebrospinal fluid samples were taken from lumbar and shunt punctures and sent for analysis and culture simultaneously (Table 1). To rule out any possible contraindication for lumbar puncture (LP), we ordered a brain computed tomography (CT) scan without contrast which showed normal results for the patient and his history.

The analysis of CSF obtained from VP shunt reservoir and spinal canal was suggestive of a bacterial shunt infection (Table 1). Considering the previous antibiotic administration, and for the coverage of both Gram-positive and Gram-negative microorganisms, we treated the patient empirically with meropenem and vancomycin.

Five days later, the result of CSF and blood cultures (Bact/Alert 3D Microbial Identification System) was reported: *Brucella* spp. We then tested the blood for Wright, Coombs Wright, 2 Mercapto Ethanol agglutination test, and anti-*Brucella* IgG (ELISA), all of which were compatible with diagnosis of brucellosis (Table 1). Since this was an unusual result, we decided to take a more detailed history from the patient's caretakers. The notable finding was consumption of unpasteurized cheese before the symptoms had started.

According to abovementioned findings, anti-*Brucella* treatment with oral rifampicin (15 mg/kg), oral trimethoprim-sulfamethoxazol (5 mg/kg trimethoprim component twice a day) and parenteral ceftriaxone (1000 mg twice a day for covering CNS infection) was started for the patient with diagnosis of VP shunt infection with *Brucella* spp. Considering our patient's condition that suffered from cerebral palsy and could not take enough care against esophagitis as an adverse effect of doxycycline (one of the first line drugs in treating brucellosis), we avoided including this medication in our regimen.

As our hospital is one of the busiest centers in the country and our patient needed intensive care before and after any surgery, it took three weeks to replace his shunt with extraventricular drain (EVD). His EVD was removed after 5 days when CSF culture was negative, and our neurosurgeons inserted a new VP shunt for the patient. Following six weeks of triple therapy, the patient was discharged on oral antibiotic therapy with trimethoprim-sulfamethoxazol and rifampicin while he was fine and symptom-free. We planned to continue antibiotic therapy for six months and to have three follow-up visits during this period, after which a lumbar puncture will be done to confirm the disease has eradicated.

## 3. Discussion

Brucellosis is a systemic infection that can involve all the body organs. Admittedly, physicians should always suspect brucellosis, particularly in endemic areas. When it comes to shunt infection in endemic communities for certain agents, namely, *Brucella*, one should consider looking for these organisms as the causal pathogen, albeit so rare and unusual. This is well advocated by available case reports [2, 4–9]. Whatever be the culprit organism, the management of VP shunt infection should include removal of the device, external drainage, parenteral antibiotics, and shunt replacement once the CSF is sterile [10].

The diagnosis of shunt infection with *Brucella* is established by isolation of the organism in CSF and/or blood cultures, abnormal CSF findings, (lymphocytic pleocytosis, high protein concentration, normal/slightly low level of glucose), and positive serology. The medications of choice for treatment of neurobrucellosis are ceftriaxone (which should be used as an initial alternative in the management of neurobrucellosis), rifampicin, doxycycline, and trimethoprim-sulfamethoxazol, as these drugs can better cross the blood-brain barrier and bind to their target receptors in the neural system [11].

As just a few cases of shunt infection with *Brucella* spp. have been observed, we report our experience. Other reported VP shunt infections with *Brucella* presented as meningitis [4, 6], fever, and abdominal pain [2, 5, 7, 8]. The last case of VP shunt infection with *Brucella* spp. was reported in 2016: an eight-year-old boy who presented with peritonitis [11]. As it can be noticed, four out of seven cases, similar to ours, presented with abdominal and gastrointestinal symptoms rather than neurologic pictures. Therefore, it is justified to look carefully for brucellosis in approach to prolonged fever with any localized symptoms. In other words, as neurobrucellosis is potentially life-threatening, the clinicians should always keep it in their differential diagnosis lists. Yet, thanks to Bact/Alert 3D Microbial Identification System, we discovered the main etiologic agent soon enough to prevent fatal sequels. The mean time of detection of microorganisms by this system has been reported between 67.8–108 h [12, 13] which is considerable compared to the conventional techniques.

A relatively confusing point in laboratory findings of our patient was neutrophylic pleocytosis in the CSF which is unusual in chronic meningitis [14]. Partially treated bacterial meningitis, Lyme disease, syphilitic meningitis, early viral and granulomatous meningitis, and leptospira meningitis are other causes of chronic meningitis with neutrophylic pleocytosis [15]. This shows that *Brucella* not only can have any clinical presentation but also can draw any laboratory picture.

Combination therapy with three or four drugs is the mainstay of neurobrucellosis treatment. However, when the type of neurobrucellosis is shunt infection, the device should temporarily be replaced with an external ventricular drain until the CSF is sterile and a new shunt could be placed. The main criteria for duration of therapy are clinical response and normal CSF values [16]. We continued oral antibiotic therapy with rifampicin and trimethoprim-sulfamethoxazol (after six weeks' combination therapy along with ceftriaxone) for our patient and will follow him clinically for six months and repeat lumbar puncture at the end of treatment.

At the end of the day, *Brucella* is here to confuse physicians by its protean manifestations. Admittedly, and because *Brucella* can potentially be fatal, physicians should think of that in approaching to every long lasting fever, irrespective of accompanying signs and symptoms. Our experience is a telling example of this, as the patient's ventriculoperitoneal shunt infection with *Brucella* manifested with constipation, fever, and abdominal pain.

## Figures and Tables

**Table 1:** Lab data during hospitalization.

Day 1		Day 3	Day 5	Day 7
ESR = 49 mm/hr CRP = 12 mg/L (negative: <5) WBC = 11300/mcl Plt = 549000/mcl HB = 11.9 g/dl		Urine culture = negative Blood culture = negative	Blood culture = *Brucella* spp. CSF sample of VP shunt = Brucella spp. CSF sample of LP = negative	Wright = 1 : 320 Coombs Wright = 1 : 640 2ME^*∗*^ = 1 : 320 Anti-*Brucella* IgG > 150
CSF analysis				
Shunt sample WBC = 100/mcl with 70% PMN Protein = 160 mg/dl RBC = 900/mcl Glucose = 20 mg/dl	LP sample WBC = 0–1/mcl RBC = 0–1/mcl Protein = 115 mg/dl Glucose = 10			

^*∗*^ *Abbreviations.* CSF: cerebrospinal fluid, WBC = white blood cells, RBC = red blood cells, LP = lumbar puncture, 2ME = 2 mercapto ethanol agglutination test.

## References

[1] Pappas G., Papadimitriou P., Akritidis N., Christou L., Tsianos E. V. (2006). The new global map of human Brucellosis. *Lancet Infectious Diseases*.

[2] Abdinia B., Barzegar M., Oskui S. (2013). Association of Brucella meningoencephalitis with cerebrospinal fluid shunt infection in a child: a case report. *Iranian Journal of Child Neurology*.

[3] Mantur B., Amarnath S., Shinde R. (2007). Review of clinical and laboratory features of human. *Indian Journal of Medical Microbiology*.

[4] Alexiou GA., llias M., Neofytus P. (2008). Ventriculoperotoneal shunt infection caused by brucella melitensis. *Pediatric Infectious Disease Journal*.

[5] Al-Otaibi A., Almuneef M., Al Shalan M. (2014). Brucella melitensis infection of ventriculoperitoneal shunt: a form of neurobrucellosis manifested as gastrointestinal symptoms. *Journal of Infection And Public Health*.

[6] Puri P., Harvey T. W. (1981). Colonization of ventriculoperitoneal shunt with brucella abortus. *British Medical Journal*.

[7] Andeson H. (1992). Unrecognized neurobrucellosis giving rise to Brucella melitensis peritonitis via a ventriculoperitoneal shunt. *European Journal of Clinical Microbiology & Infectious Diseases*.

[8] Al Shaalan M., Memish Z. A., Mahmoud S. A. (2002). Brucellosis in children: clinical observations in 115 cases. *International Journal of Infectious Diseases*.

[9] Sudhamshu K. C., Kumar A. R., Dias M. (2016). Neurobrucellosis infection of ventriculoperitoneal shunt presenting as peritonitis. *Indian Journal of Pediatrics*.

[10] Tunkel A. R., Hasbun R., Bhimraj A. (2017). Infectious disease society of American’s clinical practice guidelines for healthcare-associated ventriculitis and meningitis”. *Clinical Infectious Diseases*.

[11] Hanefi C. G., Erdam H., Gorenek L. (2008). Management of neurobrucellosis: an assessment of 11 cases. *Intenal Medicine*.

[12] Ozkurt Z., Erol S., Tasyaran M. A. (2002). Detection of Brucella melitensis by the BacT/Alert automated system and Brucella broth culture. *Clinical Microbial Infection*.

[13] Roiz M. P., Peralta F. G., Valle R. (1998). Microbiological diagnosis of brucellosis. *Journal of Clinical Microbiology*.

[14] Green J. S., Abeles S. R., Uslan D. Z. (2011). Persistent neutrophylic meningitis in an immunocompetent patient after basilar skull fracture: case report. *BMC Infectious Diseases*.

[15] Tabak F., Tanverdi M., Ozaras R. (2003). Neutophilic pleocytosis in cerebrospinal fluid: adult-onset still’s disease. *Internal Medicine*.

[16] Yetkin M., Belut C., Erdinc F. (2006). Evaluation of the clinical presentations in neurobrucellosis. *International Journal of Infectious diseases*.

